# Diallyl trisulfide attenuates metabolic syndrome *via* integrated modulation of PCSK-9/LDL-R axis, redox homeostasis, and inflammatory cytokine networks in high-carbohydrate high-fat (HCHF) diet-fed rats

**DOI:** 10.3389/fphar.2026.1781950

**Published:** 2026-05-29

**Authors:** Parvej Ahmad, Arunim Shah, Sahir Sultan Alvi, Chandra Prakash Chaturvedi, Saheem Ahmad, Bodor Bin Sheeha, Paridhi Puri, Omar W. Alothamali, M. Salman Khan

**Affiliations:** 1 Clinical Biochemistry and Natural Product Research Lab, Department of Biosciences, Integral Information and Research Center (IIRC-5), Integral University, Lucknow, Uttar Pradesh, India; 2 Department of Hematology, Stem Cell Research Center, Sanjay Gandhi Postgraduate Institute of Medical Sciences (SGPGIMS), Lucknow, Uttar Pradesh, India; 3 Department of Pharmaceutical Sciences, College of Pharmacy and Allied Health Professions, The Avera Health and Science Center, South Dakota State University, Brookings, SD, United States; 4 Department of Medical Laboratory Sciences, College of Applied Medical Sciences, University of Hail, Hail, Saudi Arabia; 5 Department of Rehabilitation Sciences, College of Health and Rehabilitation Sciences, Princess Nourah Bint Abdulrahman University, Riyadh, Saudi Arabia; 6 University Centre for Research and Development, Chandigarh University, Mohali, Punjab, India; 7 Department of Physiotherapy, College of Applied Medical Sciences, University of Ha’il, Ha’il, Saudi Arabia

**Keywords:** diallyl trisulfide (DATS), high-carbohydrate high-fat (HCHF) diet, inflammatory cytokines and chemokines, metabolic syndrome, oxidative stress, PCSK-9/LDL-R axis, rats

## Abstract

**Background:**

Metabolic syndrome (MetS), a cluster of biochemical and physiological alterations leading to the development of cardiovascular disease and type 2 diabetes (T2DM), is a major health concern worldwide. Recently, elevated level of proprotein subtilisin/kexin type-9 (PCSK-9) along with dyslipidemia, high blood pressure, blood sugar, and triglycerides have been linked to the establishment and advancement of Mets.

**Aim of this study:**

In this study, we explored the protective effects of diallyl trisulfide (DATS), a natural organosulfur compound derived from garlic, in a high-carbohydrate high-fat (HCHF) diet-induced rat model of MetS.

**Results:**

Herein, we report that DATS improves MetS parameters *via* significantly downregulating the mRNA expression of PCSK-9 (1.59-fold when compared with HCHF control) and its transcriptional activator, HNF-1α. Similarly, the mRNA expression (−3.19-fold) and the *ex-vivo* enzymatic activity of 3-hydroxy-3-methyl-glutaryl-coenzyme-A reductase (HMG-R) were attenuated in DATS-treated rats. In contrast, DATS significantly increased the low-density lipoprotein-receptor (LDL-R) mRNA expression by 1.98-fold. This unique regulation of PCSK-9/LDL-R axis, HNF1α, and HMG-R was well corroborated by the reduced plasma level of PCSK-9, low-density lipoprotein-cholesterol (LDL-C), total cholesterol (TC), and triglycerides (TGs). Furthermore, the mRNA expression and circulatory level of inflammatory cytokines *i.e*., interleukin (IL)-1, IL-6, tumor necrosis factor-α (TNF-α) and chemokines *i.e*., Chemokine (C-X-C motif) ligand (CXCL)-1, CXCL-2, and monocyte chemoattractant protein-1 (MCP-1) were also ameliorated after DATS intervention. The expression of peroxisome proliferator-activated receptors (PPAR)-α, β, and γ was also upregulated after treatment with DATS, when compared to untreated HCHF group.

**Conclusion:**

This comprehensive investigation demonstrates that DATS mitigates HCHF diet-induced MetS by targeting molecular regulators of cholesterol homeostasis, oxidative stress, glycemic indices, and inflammation. These findings suggest that DATS may serve as a promising therapeutic agent in preventing and managing MetS and associated cardiometabolic risks.

## 1 Introduction

Metabolic syndrome (MetS) is a rapidly growing disease that poses a serious health risk worldwide. The prevalence of adult individuals with MetS is estimated to be about 20%–30% in the USA, whereas ∼23% in European countries (Bhalwar, 2020; Hirode and Wong, 2020; Haverinen et al., 2021). According to the reports, the overall prevalence of the age-adjusted urban Indian population of MetS was approximately 25%. Existing evidence also illustrates that individuals with MetS have a 30%–40% increased chance of evolving Type−2 diabetes mellitus (T2DM) or cardiovascular disease (CVD) or both over a period of 20 years (Nabi et al., 2019). The collective prevalence of MetS was predicted to be 40.9% as per the National Cholesterol Education Programme (NCEP) Adult Treatment Panel III criteria in the North Indian population (Khan et al., 2018). The pathogenesis of MetS remains controversial whether different components of MetS create separate diseases or belong to a common, larger pathogenic process. Lifestyle and environmental factors, *i.e*., overeating and physical inactivity, are recognized as major contributors to MetS. High caloric intake and visceral adiposity is a major trigger that activates many MetS pathways (Pekgor et al., 2019). Overconsumption of a high-carbohydrate high-fat (HCHF) diet induces alterations in carbohydrate, fatty acid, and lipid metabolism, commencing multiple health problems, *i.e.,* atherosclerotic cardiovascular disease (ASCVD), dyslipidemia, hyperglycemia, and non-alcoholic fatty liver disease (NAFLD) (Nishikawa, 2020; Zhang et al., 2023). These risk factors also participate in the systemic progression of inflammation and oxidative stress (ElMahdy et al., 2020). Besides HCHF consumption, high fructose consumption has long been linked to metabolic health ailments, viz, obesity, alteration in lipids, inflammation, and insulin resistance (IR) (Alwahsh and Gebhardt, 2017). The level of numerous inflammatory markers like tumor necrosis factor alpha (TNFα), interleukin (IL)-6, and C-reactive protein (CRP) lead to the establishment of MetS (Islam et al., 2024; Patial et al., 2024). Moreover, IR and oxidative stress induced by obesity activate downstream inflammatory pathways, contributing to fibrosis and ASCVD (Islam et al., 2024).

Clinical and experimental investigations conducted over the last 2 decades have revealed that atherosclerosis is a low-grade, sterile, and inflammatory disease (Kong et al., 2022). Systemic and local inflammation are the major participants in the initiation and advancement of CVD. The buildup of anomalous lipoproteins like low-density lipoprotein-cholesterol (LDL-C), increased circulatory triglyceride-rich lipoproteins (TGRLs), small dense LDL cholesterol (sd-LDL-C), Apolipoprotein B (ApoB), and lower levels of antiatherogenic high-density lipoproteins cholesterol (HDL-C) are major players in the ASCVD progression (Ahmad et al., 2020; 2021; 2025). More importantly, LDL-C is primarily responsible for ASCVD establishment, therefore being a prominent target for ASCVD-related risk management (Besseling et al., 2014; Ahmad et al., 2025). The LDL-C is cleared from circulation through LDL-receptors (LDL-R) and its transcription is strictly governed through a transcription factor, sterol regulatory element binding protein 2 (SREBP-2) (Goldstein and Brown, 2015; Alvi et al., 2017b; Waiz et al., 2022). On the other hand, 3-hydroxy-3-methyl-glutaryl-coenzyme-A reductase (HMG-R), leads to the conversion of 3-hydroxy-3-methyl glutaryl-CoA (HMG-CoA) to mevalonate in the hepatocytes, which is the rate limiting step in cholesterol biosynthesis. Statins are the inhibitors of HMG-R that are used as the most privileged therapeutic molecules to achieve desired LDL-C (Goldstein and Brown, 2015; Alvi et al., 2016; Ahmad et al., 2022).

In the early 21st century, Abifadel et al. discovered and characterized proprotein convertase subtilisin/kexin type 9 (PCSK-9), revealing its crucial function in cholesterol homeostasis and initiating extensive studies in PCSK9 biology (Abifadel et al., 2014; Waiz et al., 2022). PCSK-9 engages directly with the LDL-R and facilitates its lysosomal degradation, which further limits LDL-R recycling, leading to decreased and aberrant management of LDL-C (Zhang et al., 2007; Ahmad et al., 2020). Interestingly, SREBP-2 and hepatocyte nuclear factor-1α (HNF-1α) both are well known to influence PCSK-9 expression at the transcriptional level. The regulation of both the LDL-R and PCSK-9 through a shared transcription factor, SREBP-2, undermines statins’ beneficial LDL-C reducing effects (Zhang et al., 2007; Alvi et al., 2017b; Ahmad et al., 2020; Waiz et al., 2022). In contrast, the peroxisome proliferator-activated receptors (PPARs), are (Castrillo and Tontonoz, 2004; Bougarne et al., 2018)known to regulate the glucose and lipid metabolism and inhibiting the proliferation and differentiation of adipocytes. PPARs have also been shown to affect the PCSK-9 gene expression (Castrillo and Tontonoz, 2004; Duan et al., 2012).

In India, saroglitazar, a newly accepted drug, is used to manage diabetic dyslipidemia, hyperlipidemia, hypertriglyceridemia, as well as NAFLD (Jain et al., 2015; Goyal et al., 2020). This medicine has several undesirable adverse effects, including stomach ache, nausea, vomiting, chest pain, and fever (Ratna Sai et al., 2020). Furthermore, long-term usage, cost-effectiveness, and efficacy in diverse populations must be examined. On the other hand, plant-based natural compounds have gained interest as possible innovative medicinal substances with diverse pharmacological effects (Ahmad et al., 2019; 2025; Wang et al., 2019; Nabi et al., 2020; Jahan et al., 2022; Almalki et al., 2024). In this context, natural organosulphur compounds (OSCs) such as ajoene, S-ethyl-L-cysteine (SEC), allicin, S-allyl-L-cysteine (SAC), and diallyl disulfide (DADS) have been shown to strongly inhibit the activity of carbohydrate metabolizing enzymes *i.e*., α-amylase and α-glucosidase (Ahmad et al., 2021), HMG-R (Ahmad et al., 2019; 2022), PCSK-9 (Ahmad et al., 2024), and prevention of MetS in rat model (Ahmad et al., 2025).

**FIGURE 1:**
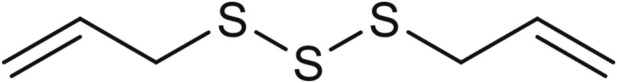
Chemical structure of diallyl trisulfide.

On the other hand, diallyl trisulfide (DATS), a structurally distinct trisulfide-containing OSC, may offer enhanced bioactivity due to its higher sulfur content, lipophilicity, and membrane permeability (Ramirez et al., 2021). Several OSCs are known to target PCSK-9 *via* HNF-1α, the potential of DATS to modulate PCSK-9 expression through both HNF-1α and HNF-4α remains largely unexplored. Moreover, distinct sulfur-rich structure of DATS (Figure 1) may confer enhanced antioxidant and anti-inflammatory potential compared to other OSCs, making it a promising candidate for broader therapeutic application in metabolic syndrome (Novakovic et al., 2024). Therefore, this study aims to comprehensively investigate the multifaceted protective role of DATS in HCHF diet-induced MetS, evaluating its impact on lipid and glycemic control, oxidative stress, inflammatory mediators, and cholesterol homeostasis gene expression. This approach not only builds upon previous findings (Ahmad et al., 2019; 2022; 2024; Novakovic et al., 2024) but also positions DATS as a potentially superior monotherapy for managing complex cardiometabolic disturbances. To our knowledge, this is the first *in vivo* study evaluating the impact of DATS on dual transcriptional regulators of PCSK-9 (HNF-1α and HNF-4α) in the context of MetS, linking it to systemic inflammatory and oxidative stress markers.

## 2 Materials and methods

### 2.1 Chemical reagents

Test compound DATS was procured from Sigma Aldrich Co., Burlington, Massachusetts, United States. Standard drug Saroglitazar (SARO), also known by Lipaglyn as a primary brand name, was obtained from Zydus Lifesciences, formerly known as Zydus Cadila Healthcare Ltd., Ahmedabad, India. ELISA kits for IL-1, IL-6, TNF-α, hs-CRP, and PCSK-9 were procured from Elabscience Biotechnology Inc., Houston, Texas United States. The RNA-Xpress™ Reagent kit was procured from HiMedia Laboratories, Mumbai, India, and the Verso cDNA Kit and Fast SYBR® Green Master Mix were obtained from Thermo Fisher Scientific Pvt. Ltd., Greater Mumbai, Maharashtra, India. Total cholesterol (TC) and TG kits were purchased from ARKRAY, Inc. Shiga, Japan. The chemicals and solvents utilized in this investigation were of analytical grade.

### 2.2 Animals

Total twenty male Sprague-Dawley (SD) rats (150–200 g and 8–9-week age) were procured from the CSIR-Central Drug Research Institute (CDRI), Lucknow. The current investigation was approved by the Institutional Animal Ethics Committee (IAEC) with approval number IU/IAEC/21/08. Rats were transported in an airconditioned vehicle and acclimatized for 7 days in the institutional animal house settings at 21–22 °C and humidity (50%–70%)-controlled environment with 12 h of light-dark cycles in polypropylene rat cages. Maximum 5 animals were housed in each cage and were fed a regular diet (chow diets) and given unlimited access to water during acclimatization of 7 days. The routine check-up was performed by an authorized veterinarian for observing hydration, nutrition, body palpation, extremities inspection, auscultation, and physical appearance every alternate day. The CPCSEA and IAEC guidelines to use animals for our study were followed throughout the study to establish strict compliance.

### 2.3 Study design

After 7 days of acclimatization, rats were randomly assigned into five different groups having four rats each group; (I) Control group; (II) HCHF, (III) DATS-1, (IV) DATS-2, and (V) standard drug (SARO). The cardiometabolic syndrome was mimicked *via* administration of 1.0 mL/rats/day of HCHF diet comprising a mixture of cholesterol 0.5% (w/v), coconut oil 3% (w/v), and cholic acid 0.25% (w/v) for 30 days. The 1.0 mL atherogenic dose was divided into two equal doses (0.5 mL) and administered at morning and evening every day along with unlimited access to drinking water supplemented with 25% fructose (w/v) (Alvi et al., 2017b; Ahmad et al., 2025). This HCHF diet was administered through intragastric intubation in all the groups except Control group in which the rats were fed with normal chow and free access to water. The intragastric intubation was chosen as it is advantageous over pelleted diet with specific compositions with test substances and offers an enhanced caloric intake and timing of exposure, ensures that each animal receives an exact, predetermined, and consistent amount of fat and cholesterol mixture, reduces the adaptive ability of rats to the feeding behaviour, higher success rates for modelling metabolic disease in rats by high fat load, bypasses the chances of animals refuse consuming specifically modified pelleted diet consisting of high amounts of specific fat types due to uncommon taste. Please refer to Table 1 for more details on experimental groups. The dosages of DATS were selected on the basis of previous studies (Singh et al., 2008; Ahmad et al., 2025). DATS was prepared in water at 1 mg/kg BW/day and 2 mg/kg BW/day, designated as DATS-1 and DATS-2, respectively. On the other hand, the standard drug, SARO, was administered at a dose of 2 mg/kg BW/rat/day as described previously (Jain et al., 2015; Ahmad et al., 2025). DATS and SARO were also administered twice a day (0.5 mL; morning and evening) through intragastric intubation in respective groups for consecutive 30 days. The rats in control group were administered with saline only.

**TABLE 1:** Study design for the induction of MetS *via* HCHF and subsequent administration of DATS and SARO in rats.

Groups	No. of rats	Treatment protocol
Control	4	Normal control; received normal rat chow and water *ad libitum*
HCHF	4	High carbohydrate high fat (HCHF) diet +25% w/v fructose dissolved in drinking water *ad libitum*
DATS-1	4	HCHF +25% w/v fructose dissolved in drinking water *ad libitum* + diallyl trisulfide (1 mg/kg BW/day)
DATS-2	4	HCHF +25% w/v fructose dissolved in drinking water *ad libitum* + diallyl trisulfide (2 mg/kg BW/day)
SARO	4	HCHF +25% w/v fructose dissolved in drinking water *ad libitum* + saroglitazar (2 mg/kg BW/day)

### 2.4 Sample collection and processing

On completion of the study (after 30 days), blood was withdrawn into a heparinized tubes *via* cardiac puncture from each rat under anaesthesia. The cervical dislocation was performed under deep anaesthesia to minimize the pain and distress in experimental animals. Desired organs were excised and either stored at −20 °C after snap freezing in liquid nitrogen or fixed with 10% neutral buffered formalin for histopathological examination.

### 2.5 Plasma lipid and lipoprotein analysis

According to the kit manufacturer’s instructions, the plasma TC, LDL-C, and HDL-C levels were determined *via* a cholesterol enzyme kit (Code No: 71LS200-60; ARKRAY, Inc., Shiga, Japan). Plasma TG level was determined through a liquid Gold TG kit (Code No: 72LS100-60; ARKRAY, Inc., Shiga, Japan) as per the manufacturer’s instructions. Plasma very low-density lipoproteins cholesterol (VLDL-C) level was assessed by the formula given by Friedewald [LDL-C = (TC)−(HDLc)−(TGs/5)] (Friedewald et al., 1972). The measurements were represented as mg/dL in plasma.

### 2.6 Assays for circulatory total antioxidant (FRAP) and paraoxonase-1 (PON-1)

A well-established and standardized protocol was employed for determining the ferric-reducing antioxidant power (FRAP) in rat plasma with some minor modifications (Wong et al., 2006). The brief methodology was previously published in our recent publication (Ahmad et al., 2025). To calculate the “total antioxidant power” concentration, a standard Ferrous sulfate was used. The protocol of Ayub *et al.* was used to determine circulatory paraoxonase-1 (PON-1) activity, and as a substrate, phenyl acetate was used (Ayub et al., 1999). The final PON-1 activity of each group was calculated through a molar extinction coefficient (MEC) of 1.31 × 10^3^ M^−1^cm^−1^ (La Du and Eckerson, 1984).

### 2.7 Measurement of plasma and hepatic lipid peroxidation products

To assess plasma and hepatic lipid peroxidation products such as cconjugated diene (CD), lipids were extracted by using Folch’s methods and also standardize in our lab by Akhter et al. (Folch et al., 1957; Akhter et al., 2019). Briefly, to extract lipids, 100 μL of plasma was mixed with a 2:1 ratio of chloroform and methanol. Captured lipids were allowed to dry under nitrogen and reconstituted in cyclohexane. The CD concentration was calculated using molar extinction coefficient (MEC) 2.52 × 10^4^ M^−1^cm^−1^. Similarly, the amount of lipid hydroperoxide (LOOH) was estimated through the Nourooz-Zadeh *et al.* method (Nourooz-Zadeh et al., 1995) and was calculated using MEC 4.3 × 10^4^ M^−1^cm^−1^. On the other hand, malondialdehyde (MDA) estimation was based on measurement of a coloured product developed by the reaction of MDA with thiobarbituric acid (TBA). In addition, plasma lipoperoxides can form malondialdehydes, which react with TBA to produce red malondialdehydes, TBA product, at high temperature. Briefly, 100 μL plasma was mixed with 1.0 mL (0.67% TBA) and 20% trichloroacetic acid (500 μL) followed by the incubation at 100 °C for 20 min. The absorbance of the supernatant was recorded at 532 nm after 12000g centrifugation for 5 min. The plasma MDA level was calculated following the Yagi method using an MEC 1.56 × 10^5^ M^−1^cm^−1^ (Yagi, 1987). Whereas the Ohkawa *et al.* procedure was employed to estimate MDA content in liver homogenate. Briefly, 10% w/v tissue homogenate was added to 0.2 mL of 8.1% SDS, 1.5 mL of 20% acetic acid (pH 3.5) and 1.5 mL of 0.8% TBA. The volume of the reaction was made up to 4 mL with distilled water and then heated on water bath at 95 °C for 60 min. After cooling under running tap water, 1.0 mL of dH_2_O and 5.0 mL of mixture of n-butanol and pyridine (15:1 v/v) were added and shaken vigorously. The absorbance of supernatant was monitored at 532 nm after centrifugation at 4,000 rpm for 10min (Ohkawa et al., 1979).

### 2.8 Measurement of SGPT and SGOT levels

The glutamate pyruvate transaminase (SGPT), also known as alanine aminotransferase (ALT), and glutamic oxaloacetic transaminase (SGOT), also known as aspartate aminotransferase (AST), were measured by SGPT (Code No. 76LS200-60) and SGOT kits (Code No: 77LS200-60) provided by ARKRAY, Inc., Shiga, Japan, as per the manufacturer’s instructions. Both SGPT and SGOT measurements were based on the IFCC- UV Kinetic method. The SGPT catalyses the transfer of the amino group from L-alanine to α-ketoglutarate resulting in the formation of pyruvate and L-glutamate. Lactate dehydrogenase then catalyses the reduction of pyruvate and simultaneous oxidation of NADH^+^ to NAD. The resulting decrease in absorbance at 340 nm is directly proportional to SGPT activity (Henry et al., 1960). Similarly, the SGOT catalyses the transfer of the amino group from aspartic acid to 2-Oxoglutarate to form oxaloacetate and L-Glutamate. The oxaloacetate thus formed reacts with 2,4 Dinitrophenyl hydrazine (2,4 DNPH) to form a corresponding hydrazone, a brownish red coloured complex in an alkaline medium. The colour intensity is directly proportional to the SGOT concentration in the sample and is measured photometrically at 505 nm. Both the SGPT and SGOT were calculated according to the manufacturer’s instructions (Reitman and Frankel, 1957).

### 2.9 Evaluation of circulatory inflammatory cytokines (IL-1, IL-6, TNF-α, MCP-1, hsCRP) and cholesterol homeostasis markers (HNF-1α and PCSK-9)

Plasma levels of TNF-α, interleukins (IL), IL-1β, IL-6, PCKS-9, HNF-1α as well as hsCRP were evaluated using the Sandwich ELISA kits from Elabscience® Texas, United States, following the manufacturer’s instructions. Briefly, 100 μL of standard, blank, and plasma of each rat from different groups was added into appropriate wells precoated with the primary antibodies against inflammatory cytokines, chemokines, PCSK-9, and HNF-1α followed by incubation for 90 min at 37 °C. 100 μL of biotinylated detection Ab working solution was then added and incubated at 37 °C for another 60 min and washed thrice with the wash buffer. 100 μL of HRP conjugate solution was added to each well and incubated for 30 min followed by five-time washings. About 90 μL of substrate was then added and incubated for 15 min and the reaction was stopped by adding 50 μL of stop solution. The plates for each plasma protein were read at 450 nm after shaking at 600 rpm on a BioRad Microplate Reader and plasma concentrations of PCKS-9, HNF-1α, MCP-1, TNF-α, IL-1β, IL-6, and hsCRP were determined *via* plotting the standard calibration curves (Ahmad et al., 2025).

### 2.10 The assessment of HMG-R activity in liver homogenate

The liver homogenate’s HMG-R enzyme activity was evaluated according to Rao and Ramakrishnan’s method with slight modifications (Venugopala Rao and Ramakrishnan, 1975; Alvi et al., 2017a). Briefly, 1 gm of fresh tissue homogenate, 9 mL of 0.1% saline arsenate (prepared in saline), and 10 mL of perchloric acid (5%) were mixed and left at room temperature for 5 minutes, then centrifuged at 2000 rpm for 10 min. From each tube, 1 mL of supernatant was withdrawn, and freshly prepared 500 μL of 1 M hydroxylamine hydrochloride aqueous solution was added. While for the analysis of the HMG-CoA level, alkaline hydroxylamine hydrochloride (500 μL) was mixed and stirred. The reaction was incubated at room temperature for 5 min and then 1.5 mL of 0.616 M ferric chloride containing 5.2% TCA (prepared in 0.65N HCl) was added, mixed well, and left at for another 10 min at room temperature. The absorbance was taken at 540 nm against a reagent blank using a Bio-spectrophotometer kinetics (Eppendorf, Hamburg, Germany).

### 2.11 Hepatic gene expression analysis

As previously described, the quantitative real-time polymerase chain reaction (qRT-PCR) method was employed to assess the expression of particular genes involved in the cholesterol homeostasis and inflammatory cascades (Nabi et al., 2023).

#### 2.11.1 RNA extraction from hepatic tissue

The total cellular RNA (tcRNA) from liver tissue was isolated by using the RNA-Xpress™ reagent kit from HiMedia, Maharashtra, India, following the manufacturer’s instructions. The detailed methodology for tcRNA extraction is available in a previously published article (Alvi et al., 2017a).

#### 2.11.2 Reverse transcription of the tcRNA

The complementary DNA (cDNA) was synthesized through reverse transcription of tcRNA *via* a high-capacity cDNA Reverse Transcription Kit, Thermo Fisher Scientific, Mumbai, India, following the manufacturer’s guidelines. The detailed method was explained in our previous report (Ahmad et al., 2025).

#### 2.11.3 qRT-PCR analysis

To evaluate the gene expression analysis, previously prepared cDNA was diluted to 5 ng/μL (final concentration). The oligonucleotide primers that were used in this experiment are mentioned in Supplementary Table S1. The qRT-PCR was carried out according to our previously published article (Alvi et al., 2017a). The max volume of each qRT-PCR reaction was 20 μL comprising of cDNA, forward and reverse primer (200 nM each) and 2X Fast SYBR® Green Master Mix (10 μL) from Thermo Fisher Scientific, Pvt. Ltd., Mumbai, India. The house keeping gene, β-actin, was used for normalization and the fold change in gene expression was calculated using 2^−ΔΔCT^ method (Livak and Schmittgen, 2001; Ahmad M. et al., 2026).

### 2.12 Enzymatic and non-enzymatic antioxidant function in liver

The catalase (CAT) and superoxide dismutase (SOD) enzyme activities in liver post mitochondrial supernatant (PMS) were measured according to the well-established methods (Sinha, 1972; Kakkar et al., 1984). The glutathione peroxidase (Gpx) activity was determined using the method of Hafeman et al. (1974), the glutathione-S-transferase (GST) activity in liver PMS fraction was assessed by the procedure of Habig et al. (Habig et al., 1974), and glutathione reductase (Gred) activity in liver PMS was performed through previously published and standardized methods (Hashim et al., 2019). The non-enzymatic antioxidant, reduced glutathione (GSH) content of liver homogenate was estimated through Ellman’s method with slight modification (Ellman, 1959; Nabi et al., 2023).

### 2.13 Histopathological studies

The neutral buffered formalin (10%) was used to fix the excised liver for histological examination. The fixed liver was rinsed under running water and dehydrated using an ethyl alcohol gradient to avoid excessive distortion of the tissue. The tissues were cleared with xylene to remove residual ethanol and substantial amount of fat and then left for wax infiltration and subsequently embedded to form paraffin blocks. The paraffin blocks were sectioned through a microtome to produce 4–5 μm thick sections. These slides were then stained with hematoxylin-eosin and were subsequently observed and photographed to investigate histo-architectural modifications in liver tissues as described recently (Ahmad M. et al., 2026).

### 2.14 Protein estimation

The content of the proteins in all the samples was quantified using the Pierce BCA Protein assay kit, Thermo Fisher Scientific, Greater Mumbai, India, and a calibration curve was drawn using bovine serum albumin as the standard protein (Ahmad M. et al., 2026).

### 2.15 Data analysis

In all molecular and biochemical tests, samples were taken in triplicate, and the results were expressed as Mean ± SEM. The statistical significance was evaluated by using one-way analysis of variance (ANOVA) followed by the Post Hoc Tukey-Kramer multiple comparisons test by using GraphPad Prism version 8.4.0 (Ahmad et al., 2025).

## 3 Results

### 3.1 DATS treatment ameliorates vascular lipid levels in HCHF-induced cardiometabolic syndrome in a murine model

**FIGURE 2:**
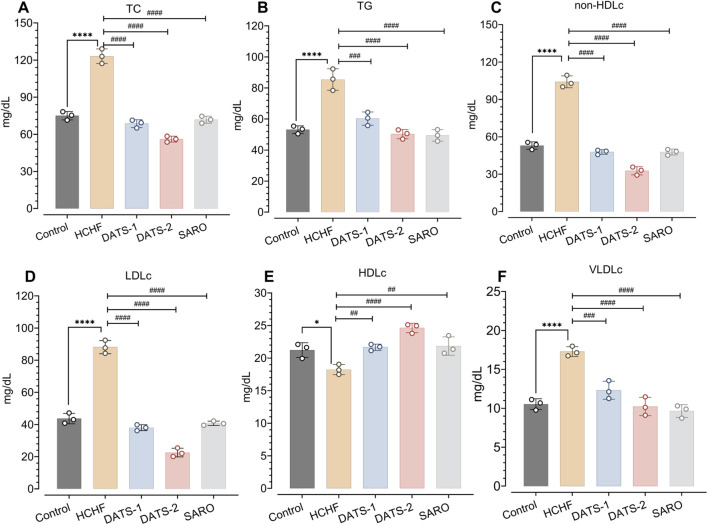
Diallyl trisulfide (DATS) ameliorates vascular lipid and lipoprotein levels in HCHF-induced cardiometabolic syndrome in rats. **(A–F)** The level of plasma TC, TG, non-HDLc, LDLc, HDLc, and VLDLc. Values are expressed as mean (mg/dL) ± SEM from the plasma of four rats in each group. Control: Normal control; HCHF, High-carbohydrate high-fat diet-induced cardiometabolic syndrome control; DATS-1 and DATS-2, Treatment with DATS at doses of 1 mg and 2 mg/kg BW/rat/day, respectively; saroglitazar (SARO): Treatment with standard drug SARO at a dose of 2 mg/kg BW/rat/day. The statistical significance was evaluated by using one-way analysis of variance (ANOVA) followed by the Post Hoc Tukey-Kramer multiple comparisons test by using GraphPad Prism version 8.4.0. The statistical significance has been denoted in terms of p-value in each panel across the figures. Significantly different from control at ****p < 0.0001. Significant from control at *p < 0.05. Significantly different from HCHF at ^####^p < 0.0001. Significantly different from HCHF at ^###^p < 0.001. Significantly different from HCHF at ^##^p < 0.01. Significantly different from HCHF at ^#^p < 0.05.

After 4 weeks of therapy, the cardioprotective potential of DATS was demonstrated in HCHF-induced cardiometabolic syndrome rats. Induction of cardiometabolic syndrome with HCHF diet drastically altered vascular cholesterol levels *i.e.,* TC, TG, non-HDL-C, LDL-C, and VLDL-C in HCHF control rats. Briefly, the circulatory TC and TG levels markedly rose from 75.03 to 53.13 mg/dL, respectively, in the Control rats to 123.1 mg/dL (+64.07%; p < 0.0001) and 85.48 mg/dL (+60.89%; p < 0.0001), respectively, in HCHF rats. Similarly, non-HDL-C, LDL-C, and VLDL-C levels were significantly (p < 0.0001) higher in HCHF rats (+96.32%, +101.60%, and +64.19%, respectively), in contrast to the Control rats (Figure 2). Subsequent treatment with DATS exhibited a significant amelioration in TC, TG, non-HDL-C, LDL-C, and VLDL-C levels. Both the DATS doses (DATS-1 and DATS-2) lowered the TC, TG, LDL-C, VLDL-C, and non-HDL-C levels, with maximum restoration observed in DATS-2-treated rats by −54.40% (p < 0.0001), −41.21% (p < 0.0001), −74.40% (p < 0.0001), −40.80% (p < 0.0001), and −68.91% (p < 0.0001), respectively, relative to the Control group. In contrast, standard drug SARO-treated rats also showed a decline in TC, TG, LDL-C, VLDL-C, and non-HDL-C levels by −41.68%, −42.12%, −53.71%, −44.19%, and −54.05% (p < 0.0001), respectively, when compared to HCHF rats. In contrast, vascular HDL-C level was reduced from 21.23 mg/dL in the Control rat to 18.24 mg/dL (−14.08%; (p < 0.05)) in HCHF rats. After subsequent treatment with DATS (DATS-1 and DATS-2) for 4 weeks, the HDL-C levels were markedly enhanced in both groups, with the highest restoration of +23.74% observed in higher doses of DATS (p < 0.0001), as compared to HCHF rats. While only a +19.79% (p < 0.01) increment was observed in the HDL-C level in the SARO-treated group as compared to the HCHF group (Figure 2).

### 3.2 DATS administration ameliorates the lipid ratios in cardiometabolic syndrome rats

The vascular lipid ratios, i.e., LDL-C/HDL-C and HDL-C/TC, have been considered substantial predictors of both the severity and prevalence of CVD. In the present study, HDL-C/LDL-C, HDL-C/TC, TC/HDL-C, and LDL-C/HDL-C ratios were measured using the data portrayed in Figure 2. The data obtained from the lipid profile suggested that the ratios of HDL-C/LDL-C and HDL-C/TC were markedly reduced to 2.35 (p < 0.0001) and 1.91 folds (p < 0.0001), respectively, while the lipid ratio of TC/HDL-C, as well as LDL-C/HDL-C, was found to be significantly elevated by 1.91 (p < 0.0001) and 2.35 (p < 0.0001) folds, respectively, in the HCHF rats relative to the Control rats (Table 2). Administration of DATS-2 showed a marked improvement in HDL-C/LDL-C and HDL-C/TC ratios by 4.91 (p < 0.0001) and 2.76 (p < 0.0001) folds, reactively, as well as a diminution was observed of 2.76 (p < 0.0001) and 4.92 folds (p < 0.0001) in TC/HDL-C and LDL-C/HDL-C ratios, respectively, relative to corresponding HCHF rats. However, SARO also exhibited a significant increase of 2.59 and 2.05 folds in HDL-C/LDL-C and HDL-C/TC ratios, respectively, although a reduction of 2.05 and 2.59 folds was observed in TC/HDL-C and LDL-C/HDL-C ratios, respectively, compared to HCHF rats.

**TABLE 2:** Effect of DATS and SARO treatment on lipid ratios.

Groups	HDL-C/LDL-C	HDL-C/TC	TC/HDL-C	LDL-C/HDL-C
Control	0.486 ± 0.025	0.283 ± 0.020	3.534 ± 0.130	2.059 ± 0.058
HCHF	0.207 ± 0.015 (−2.35 f)^****^ ^(p= <0.0001)^	0.148 ± 0.010 (−1.91 f)^****^ ^(p= <0.0001)^	6.749 ± 0.170 (+1.91 f)^**** (p= <0.0001)^	4.832 ± 0.136 (+2.35 f)^****^ ^(p= <0.0001)^
DATS-1	0.508 ± 0.029 (+2.45 f)^####^ ^(p= <0.0001)^	0.282 ± 0.022 (+1.91 f)^####^ ^(p= <0.0001)^	3.549 ± 0.125 (−1.90 f)^####^ ^(p= <0.0001)^	1.968 ± 0.054 (−2.46 f) ^####^ ^(p= <0.0001)^
DATS-2	1.017 ± 0.050 (+4.91 f)^####^ ^(p= <0.0001)^	0.409 ± 0.028 (+2.76 f)^####^ ^(p= <0.0001)^	2.445 ± 0.082 (−2.76 f)^####^ ^(p= <0.0001)^	0.983 ± 0.050 (−4.92 f) ^####^ ^(p= <0.0001)^
SARO	0.536 ± 0.032 (+2.59 f)^####^ ^(p= <0.0001)^	0.304 ± 0.024 (+2.05 f)^####^ ^(p= <0.0001)^	3.286 ± 0.117 (−2.05 f) ^####^ ^(p= <0.0001)^	1.867 ± 0.058 (−2.59 f) ^####^ ^(p= <0.0001)^

Values are mean ± SEM, from the plasma of four rats in each group.

‘f’ stands for fold change observed.

### 3.3 DATS treatment controls glycaemic indices in HCHF-induced cardiometabolic syndrome in rats

After feeding the HCHF diet daily for 4 weeks, the fasting blood glucose (FBG) level was markedly increased by 87.5% (p < 0.0001) in HCHF group in contrast to the control group. While subsequent administration of DATS displayed a significant decrease in FBG level, with the maximum diminution of 17.99% (p < 0.0001) measured in the DATS-2 group as compared to the untreated HCHF rats. Whereas, standard drug, SARO-treated rats also showed a significant decline in the level of FBG by 23.21% (p < 0.0001) compared to the HCHF group (Table 3). Insulin facilitates glucose absorption from the bloodstream, affecting carbohydrate, lipid, and protein metabolism. The insulin level was markedly decreased by 23.84% (p < 0.0001) in the HCHF group compared to the Control rats. However, DATS administration slightly, but not significantly (p > 0.05), improved the insulin level by 4.05% and 6.16% in DATS-1 and DATS-2 treated groups, respectively, when compared to HCHF rats. In contrast, treatment with SARO markedly increased the insulin level by 38.34% (p < 0.0001), when compared to HCHF rats. Glycated hemoglobin (HbA1c) is a form of hemoglobin that is chemically linked to sugar and is a major predictor and a prime biomarker of diabetes (Gilstrap et al., 2019). For this reason, we also estimated the HbA1c level and observed a marked increase of HbA1c (67.13%; p < 0.0001) in the HCHF group as compared to the Control rats. This elevated HbA1c level was significantly reduced in DATS and SARO-treated rats. The maximum reduction (14.26%; p < 0.0001) was observed in DATS-2-treated rats, which was even higher (11.82%; p < 0.0001) than in SARO-treated rats, in contrast to HCHF rats. Also, abundant plasma creatinine is an established indicator in the development of diabetic complications, specifically nephropathy (Elsayed et al., 2019; Waiz et al., 2023). Results observed from our interventional groups, after HCHF consumption, the creatinine level was significantly elevated by 129.32% (p < 0.0001) when compared to the control group. This escalation in creatinine levels was considerably reversed by 45.74% (p < 0.0001) and 52.91% (p < 0.0001) after treatment with DATS-1 and DATS-2, respectively, as compared to the HCHF group. The treatment with the standard drug SARO also exhibited a significant reduction in creatinine level (58.74%), comparable to HCHF rats.

**TABLE 3:** DATS and SARO alleviate glycemic profile in HCHF-induced cardiometabolic syndrome in rats.

Groups	Fasting blood glucose (mg/dL)	Insulin (μU/mL)	HbA1c (%)	Creatinine (mg/dL)
Control	80.64 ± 2.528	1.493 ± 0.055	3.653 ± 0.017	0.614 ± 0.018
HCHF	151.2 ± 2.81 (+87.5%)^**** (p= <0.0001)^	1.137 ± 0.020 (−23.84%)^**** (p= <0.0001)^	6.127 ± 0.017 (+67.13%)^**** (p= <0.0001)^	1.408 ± 0.019 (+129.32%)^**** (p= <0.0001)^
DATS-1	134.5 ± 2.21 (−11.04%)^##^ ^(p= 0.0031)^	1.183 ± 0.020 (+4.05%)^ns (p=0.8003)^	5.687 ± 0.049 (−7.17%)^#### (p= <0.0001)^	0.764 ± 0.015 (−45.74%)^#### (p= <0.0001)^
DATS-2	124.0 ± 2.08 (−17.99%)^#### (p= <0.0001)^	1.207 ± 0.023 (+6.16%)^ns^ ^(p= 0.4973)^	5.253 ± 0.026 (−14.26%)^#### (p= <0.0001)^	0.663 ± 0.014 (−52.91%)^#### (p= <0.0001)^
SARO	116.1 ± 2.50 (−23.21%)^#### (p= <0.0001)^	1.573 ± 0.010 (+38.35%)^#### (p= <0.0001)^	5.403 ± 0.031 (−11.82%)^#### (p= <0.0001)^	0.581 ± 0.005 (−58.74%)^#### (p= <0.0001)^

Values are represented as mean ± SEM, from the plasma of four rats in each group.

### 3.4 DATS treatment helps maintain liver health

**FIGURE 3:**
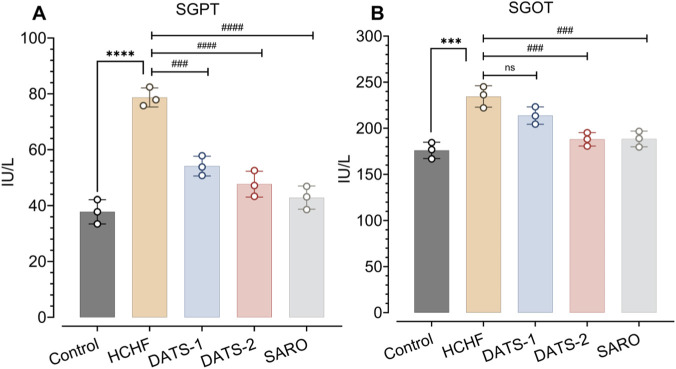
Treatment with DATS and SARO restores the hepatic functionality in HCHF-induced MetS in rats. **(A)** The level of serum glutamate pyruvate transaminase (SGPT), also known as alanine aminotransferase (ALT). **(B)** The level of serum glutamic-oxaloacetic transaminase (SGOT), also known as aspartate aminotransferase (AST). Values are mean (IU/L) ±SEM from the plasma of four rats in each group. The statistical significance was evaluated by using one-way ANOVA followed by the Post Hoc Tukey-Kramer multiple comparisons test by using GraphPad Prism version 8.4.0. Significantly different from control at ****p < 0.0001; Significantly different from control at ***p < 0.001; Significantly different from HCHF at ^####^p < 0.0001; Significantly different from HCHF at ^###^p < 0.001; Non-significant from HCHF at ^ns^p>0.05.

SGOT and SGPT are enzymes found in the liver that are used to measure liver health. The SGOT and SGPT are released into the bloodstream when liver cells are damaged or inflamed. The HCHF diet significantly increased the circulatory SGPT and SGOT levels by 111.35% (p < 0.0001) and 33.24% (p < 0.001), respectively, compared to the Control group. On the other hand, DATS administration significantly reduced SGPT and SGOT levels in a dose-dependent manner when compared to the HCHF rats. The maximum restoration was observed in DATS-2-treated rats by 39.20% (p < 0.0001) and 19.84% (p < 0.001) in SGPT and SGOT levels, respectively, compared to HCHF rats. On the other hand, SARO treatment also diminished the SGPT and SGOT levels by 46.33% (p < 0.0001) and 19% (p < 0.001), respectively, as compared to HCHF rats. Here, it is interesting to note that both the drugs, DATS-2 and SARO, equally reduced the SGOT level in the interventional group (Figures 3A,B).

### 3.5 DATS treatment lowers plasma PCSK-9 and HNF-1α levels in the interventional group

**FIGURE 4:**
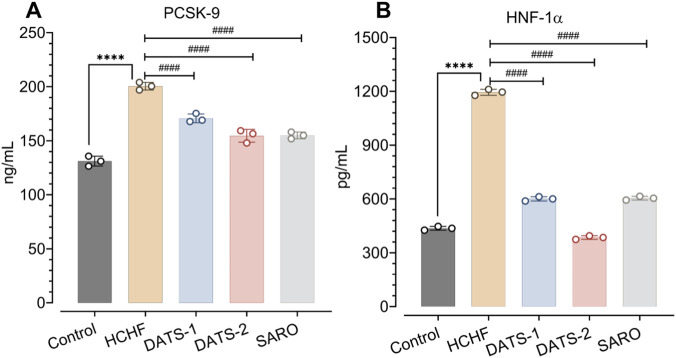
DATS and SARO treatment decrease the circulatory proprotein convertase subtilisin/kexin type-9 (PCSK-9) and its transcriptional activator HNF1α in HCHF-induced cardiometabolic syndrome in rats. **(A)** The level of PCSK-9 in HCHF-induced cardiometabolic syndrome in rats. **(B)** The level of plasma HNF1α in HCHF-induced cardiometabolic syndrome in rats. Values are mean (ng/mL) ±SEM from the plasma of four rats in each group. The statistical significance was evaluated by using one-way ANOVA followed by the Post Hoc Tukey-Kramer multiple comparisons test by using GraphPad Prism version 8.4.0. Significantly different from control at ****p < 0.0001; Significantly different from HCHF at ^####^p < 0.0001.

PCSK-9 is a renowned influencer that uplifts the plasma LDL-C level, as it interacts with the LDL-R and promotes its lysosomal degradation (Horton et al., 2007; Alvi et al., 2017b). The PCSK-9 expression is primarily regulated through HNF-1α and HNF-4α, which act as transcriptional factors of PCSK-9 (Ai et al., 2012; Alvi et al., 2017a; Ahmad et al., 2020). Our study found that consumption of HCHF diet markedly elevated circulatory levels of PCSK-9 and HNF-1α by 52.74% (p < 0.0001) and 173.99% (p < 0.0001), respectively, relative to the control group. Simultaneous treatment with DATS-2 showed a significant reduction of elevated plasma PCSK-9 levels by 22.20% (p < 0.0001) and HNF-1α levels by 67.74% (p < 0.0001) relative to HCHF rats. In addition, a marked reduction of 21.6% (p < 0.0001) in plasma PCSK-9 and 49.48% (p < 0.0001) in HNF-1α levels in SARO-treated rats was also observed when compared to HCHF rats. This deterioration in the circulatory PCSK-9 protein level may be responsible for restoring the LDL-C levels in the DATS and SARO treated groups in our study, as reducing the circulatory PCSK-9 level is well known to increase the LDL-C clearance *via* enhanced number of available LDL-Rs on hepatocytes (Goldstein and Brown, 2015; Ahmad et al., 2020) (Figures 4A,B).

### 3.6 Treatment with DATS deteriorates the plasma inflammatory cytokines and chemokines in HCHF-induced cardiometabolic syndrome in rats

**FIGURE 5:**
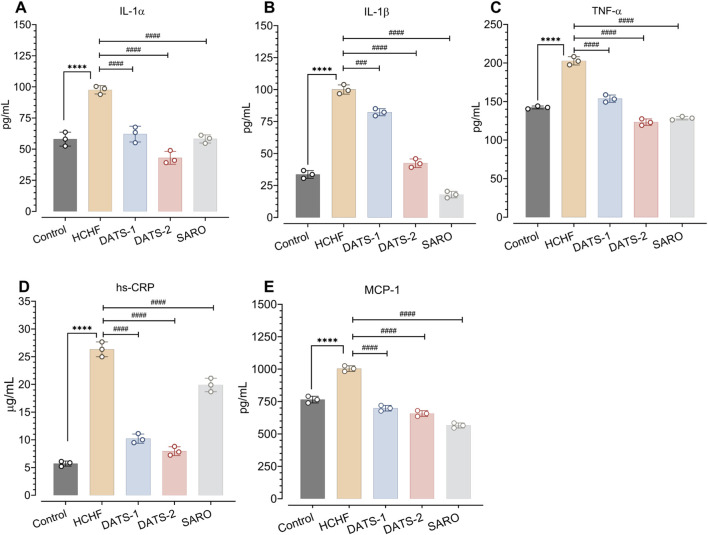
Treatment with DATS and SARO ameliorate the circulatory inflammatory markers. **(A, B)** The level of IL-1α and IL-1β, respectively, in HCHF-challenged cardiometabolic syndrome in rats. **(C)** The level of TNF-α. **(D)** The level of high-sensitivity C-reactive protein (hs-CRP). **(E)** The level of monocyte chemoattractant protein-1 (MCP-1). Values are represented in mean (pg/mL and µg/mL) ±SEM from the plasma of four rats in each group. The statistical significance was evaluated by using one-way ANOVA followed by the Post Hoc Tukey-Kramer multiple comparisons test by using GraphPad Prism version 8.4.0. Significantly different from control at ****p < 0.0001; Significantly different from HCHF at ^####^p < 0.0001; Significantly different from HCHF at ^###^p < 0.001.

The initiation, propagation, and establishment of atheroma and the ensuing consequences for cardiovascular health have all been extensively associated with inflammation (Wang et al., 2019; Ahmad et al., 2020). Different inflammatory mediators like IL-1α, IL-1β, and TNF-α are known to fuel the progress of fatty streak formation and atherosclerotic plaques (Evans et al., 2022; Waiz et al., 2022). From our *in vivo* study, HCHF-diet-induced cardiometabolic syndrome in rats showed a marked increase in the level of these circulatory inflammatory mediators compared to the Control rats. The circulatory IL-1α, IL-1β, and TNF-α levels were observed to significantly (p < 0.0001) increase by 68.83%, 195.48%, and 42.23%, respectively, in the HCHF rats in contrast to the Control rats. These elevated circulatory levels of IL-1α, IL-1β, and TNF-α were restored to the normal level after DATS treatment in a dose-dependent manner, with the maximum restoration observed in DATS-2-treated rats by 55.91% (p < 0.0001), 57.62% (p < 0.0001), and 39.23% (p < 0.0001), respectively, relative to the HCHF rats. Whereas standard drug SARO-treated rats also showed a deterioration of circulatory levels of IL-1α, IL-1β, and TNF-α by 40.46% (p < 0.0001), 82.05% (p < 0.0001), and 36.51% (p < 0.0001), respectively, relative to HCHF rats (Figures 5A–C).

In the same way, it is widely accepted that CRP plays a significant role in inflammation-induced CVD and is a key diagnostic biomarker. Results from our study established that the circulatory high-sensitivity (*hs)*-CRP level was raised by 361.93% (p < 0.0001) in the HCHF group relative to the control rats. Subsequent treatment with DATS deteriorated the elevated *hs*-CRP level, with the maximum deterioration observed in DATS-2-treated rats by 69.69% (p < 0.0001) relative to HCHF rats. In contrast, administration with SARO lessened the *hs*-CRP level by 24.42% (p < 0.0001); this was comparatively less than the value observed in the group receiving DATS (Figure 5D). Moreover, several publications have demonstrated the importance of monocyte chemotactic protein-1 (MCP-1) in triggering the development of atherosclerotic plaque by drawing and recruiting monocytes to the site of endothelial damage (Ahmad et al., 2020; Waiz et al., 2022). Similarly, we also observed that the circulatory MCP-1 level significantly increased by 31.17% (p < 0.0001) in the HCHF rats, relative to the Control group. Simultaneous administration with DATS markedly reduced the elevated level of MCP-1 in a dose dose-dependent manner, with the maximum reduction observed in DATS-2 treated rats by 34.47% (p < 0.0001), thus indicating a defensive role of DATS towards the progression of atheromatous plaque and consequent CVD events (Figure 5E).

### 3.7 Treatment of DATS recovers the circulatory FRAP and PON-1 function

**FIGURE 6:**
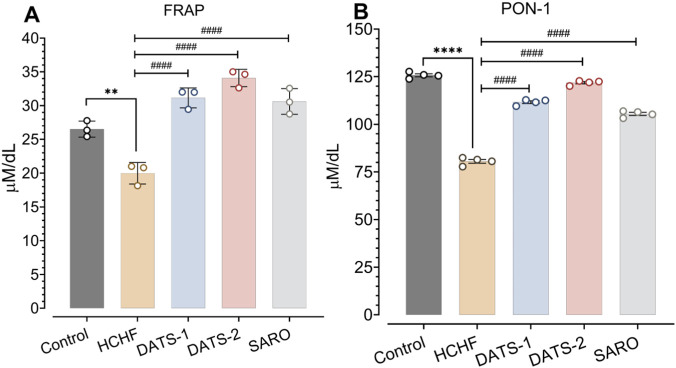
Treatment with DATS and SARO improves the plasma antioxidant status. **(A)** The level of FRAP in HCHF-induced MetS in rats. **(B)** The level of HDL-associated paraoxonase-1 (PON-1) activity in HCHF-diet induced metabolic stress in rats. The values are represented in Mean (μmol/dL) ±SEM of four rats in each group. The statistical significance was evaluated by using one-way ANOVA followed by the Post Hoc Tukey-Kramer multiple comparisons test by using GraphPad Prism version 8.4.0. Significantly different from control at ****p < 0.0001; Significantly different from control at **p < 0.01; Significantly different from HCHF at ^####^p < 0.0001.

The HCHF diet significantly decreased the circulatory FRAP and PON-1 levels by 24.63% (p < 0.01) and 35.93% (p < 0.0001), respectively, relative to the Control rats. However, simultaneous administration with DATS increased the FRAP and PON-1 levels in a dose-dependent manner, with peak improvement of 70.57% (p < 0.0001) and 51.21% (p < 0.0001), respectively, in DATS-2-treated groups when compared to the HCHF rats. Whereas SARO treatment also demonstrated significant (p < 0.0001) improvements in FRAP and PON-1 levels by 35.25% and 30.97%, respectively, relative to HCHF rats (Figures 6A,B).

### 3.8 Treatment with DATS and SARO modulates *in-vivo* hepatic HMG-R activity

**FIGURE 7:**
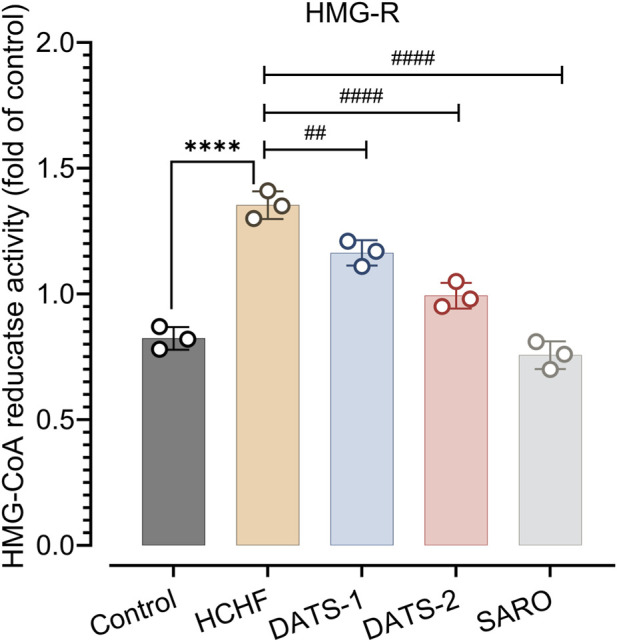
Modulation of *ex-vivo* hepatic 3-hydroxy-3-methylglutaryl coenzyme-A reductases (HMG-R) activity by DATS and SARO in HCHF-challenged rats. The values are represented as fold change in HMG-R activity. The *ex-vivo* HMG-R activity was measured as the 3-hydroxy-3-methylglutaryl coenzyme-A (HMG-CoA) to mevalonate ratio, where the lower the ratio, the stronger the enzymatic activity. The statistical significance was evaluated by using one-way ANOVA followed by the Post Hoc Tukey-Kramer multiple comparisons test by using GraphPad Prism version 8.4.0. Significantly different from control at ****p < 0.0001; Significantly different from HCHF at ^####^p < 0.0001; Significantly different from HCHF at ^##^p < 0.01.

It is well known that a high-cholesterol diet has been linked to increased hepatic HMG-R activity through altered SREBP-2 processing (Alvi et al., 2017b; Waiz et al., 2022). Likewise, in our study, the HCHF diet-induced MetS in rats shows a notable 1.65-fold increase (p < 0.0001) in the *in-vivo* hepatic HMG-R activity in HCHF rats relative to control rats (Figure 7). Administration of the test compound, DATS, with two different doses, DATS-1 and DATS-2, showed significant inhibition of 1.15 (p < 0.01) and 1.36-fold (p < 0.0001), respectively, in hepatic HMG-R activity when compared to the HCHF rats. Additionally, compared to rats given HCHF, the SARO group’s hepatic HMG-R activity was reported to decrease by 1.78-fold (p < 0.0001).

### 3.9 DATS restores the expression of cholesterol metabolism related hepatic genes

**FIGURE 8:**
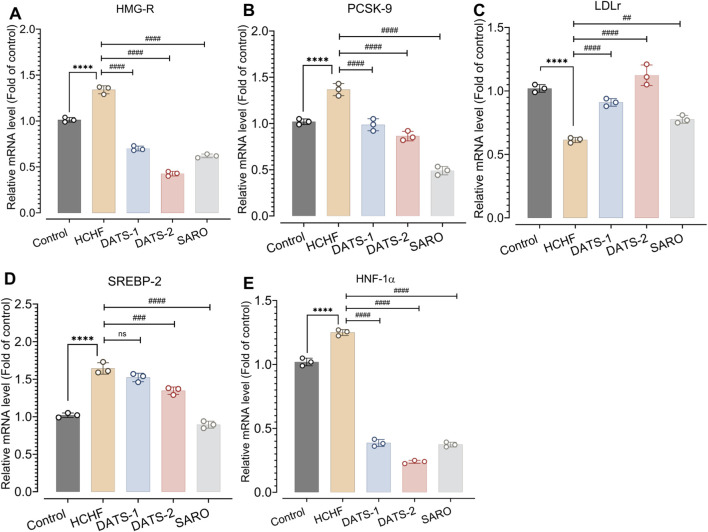
The treatment with DATS and SARO regulates the expression of genes related to cholesterol homeostasis. **(A–E)** The mRNA expression of HMG-R, PCSK-9, LDLr, SREBP-2, and HNF1α, respectively, in HCHF-induced cardiometabolic syndrome in rats. Each bar depicts the Mean ± SEM fold variation in relative mRNA level of three independent experiments. The statistical significance was evaluated by using one-way ANOVA followed by the Post Hoc Tukey-Kramer multiple comparisons test by using GraphPad Prism version 8.4.0. Significantly different from control at ****p < 0.0001; Significantly different from HCHF at ^####^p < 0.0001; Significantly different from HCHF at ^###^p < 0.001; Significantly different from HCHF at ^##^p < 0.01; Non-significant from HCHF at ^ns^p>0.05.

Hepatic genes like HMG-R, PCSK-9, LDL-R, SREBP-2, and HNF-1α engage in cholesterol homeostasis and rigorously control the pathway of cholesterol synthesis (Alvi et al., 2017b; Ahmad et al., 2020). Results from our *in vivo* study displayed that a high cholesterol diet significantly (p < 0.0001) elevated the hepatic gene expression of HMG-R (1.34-fold), PCSK-9 (1.37-fold), SREBP-2 (1.64-fold), and HNF-1α (1.25-fold), relative to the control rats. In contrast, HCHF rats showed a 1.64-fold (p < 0.0001) downregulation in hepatic LDL-R mRNA expression. Subsequent treatment with DATS (DATS-1 and DATS-2) significantly reduced these over-expressed genes in a dose-dependent manner, with the maximum restoration observed in DATS-2-treated rats. DATS-2 treatment showed a marked reduction in hepatic mRNA expression of HMG-R, PCSK-9, SREBP-2, and HNF-1α levels by 3.19 (p < 0.0001), 1.59 (p < 0.0001), 1.21 (p < 0.001), and 5.32-fold (p < 0.0001), respectively, relative to HCHF rats. Whereas, standard drug SARO-treated rats exhibit a marked reduction (p < 0.0001) of HMG-R, PCSK-9, SREBP-2, and HNF-1α mRNA expression levels by 2.2, 2.8, 1.84, and 3.36 folds, respectively, relative to HCHF rats. In contrast, DATS treatment significantly upregulated the LDL-R mRNA expression, with the maximum improvement of 1.98-fold observed in DATS-2 treated rats (p < 0.0001), as well as treatment with SARO also enhanced the LDL-R mRNA expression by 1.26-fold (p < 0.01), relative to HCHF rats (Figures 8A–E).

### 3.10 DATS treatment downregulates the expression of inflammatory cytokines and chemokines

**FIGURE 9:**
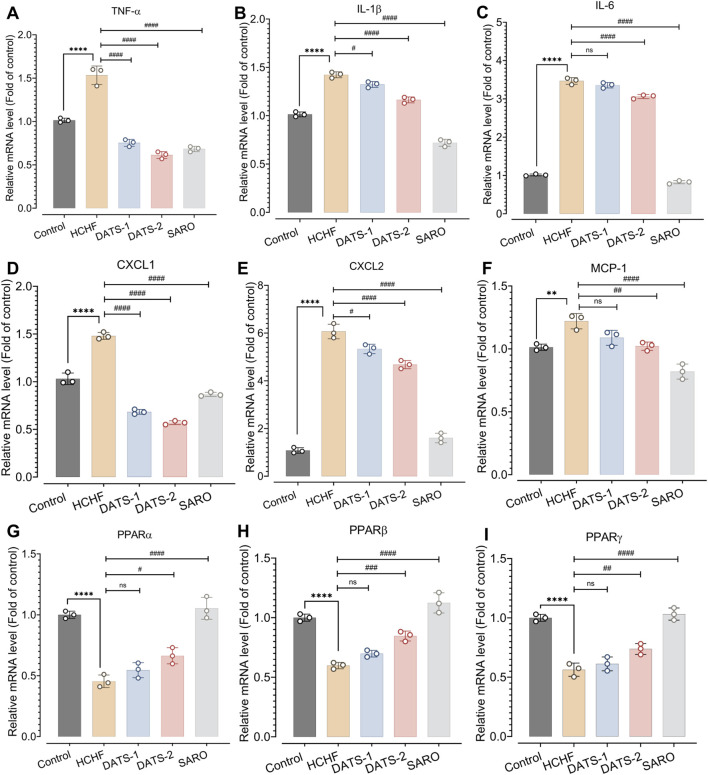
Impact of DATS and SARO treatment on gene expression patterns of selected hepatic inflammatory and peroxisome proliferator-activated receptors (PPARs) involved in lipid and glucose homeostasis in HCHF-induced cardiometabolic syndrome in rats. **(A)** Relative mRNA expression of TNF-α. **(B)** Relative mRNA expression of IL-β. **(C)** Relative mRNA expression of IL-6. **(D)** Relative mRNA expression of CXCL-1. **(E)** Relative mRNA expression of CXCL-2. **(F)** Relative mRNA expression of MCP-1. **(G)** Relative mRNA expression of PPARα. **(H)** Relative mRNA expression of PPAR β. **(I)** Relative mRNA expression of PPAR γ. Each bar shows the mean ± SEM relative mRNA level (fold of control) from three independent experiments. The statistical significance was evaluated by using one-way analysis of variance (ANOVA) followed by the Post Hoc Tukey-Kramer multiple comparisons test by using GraphPad Prism version 8.4.0. Significantly different from control at ****p < 0.0001; Significantly different from control at **p < 0.01; Significantly different from HCHF at ^####^p < 0.0001; Significantly different from HCHF at ^###^p < 0.001; Significantly different from HCHF at ^##^p < 0.01; Significantly different from HCHF at ^#^p < 0.05; Non-significant from HCHF at ^ns^p>0.05.

Some inflammatory representatives, like TNF-α, IL-1, IL-6, CRP, CXCL1, CXCL2, and MCP-1, have been identified as important mediators in the progression and development of atherosclerotic events (Evans et al., 2022; Xing and Lin, 2025). That is why we also evaluate these markers in the liver. Results from our interventional study exhibited that TNF-α, IL-1β, and IL-6 mRNA expression were significantly (p < 0.0001) upregulated in HCHF rats by 1.53, 1.42, and 3.46-fold, respectively, relative to the Control group. These markers were downregulated in a dose-dependent manner, with the highest downregulation of 2.50, 1.22, and 1.13-fold, respectively, in the DATS-2 administered group relative to the HCHF group (p < 0.0001). While treatment with the standard drug SARO also displayed a significant (p < 0.0001) downregulation of 2.25, 1.98, and 4.22-fold in TNF-α, IL-1β, and IL-6 mRNA expression, respectively, relative to HCHF rats (Figures 9A–C). Similarly, overexpression of 1.48 (p < 0.0001), 6.06 (p < 0.0001), and 1.22 (p < 0.01)-fold was observed for inflammatory chemokines CXCL1, CXCL2, and MCP-1 mRNA, respectively, in HCHF rats when compared to the control group. For two dosages of DATS were used, DATS-2 treatment exhibited higher restoration of 2.60 (p < 0.0001), 1.29 (p < 0.0001), and 1.19 (p < 0.01) folds in CXCL1, CXCL2, and MCP-1 mRNA expression levels, respectively, relative to HCHF rats (Figures 9D–F).

### 3.11 DATS treatment enhances PPAR gene expression

The PPARs are ligand-activated transcription factors that control key metabolic processes, along with lipid and glucose homeostasis and genes involved in cell differentiation. PPAR-α, PPAR-β, and PPAR-γ were analysed for hepatic gene expression. Results from our *in-vivo* study displayed marked (p < 0.0001) downregulation in PPAR-α, β, and γ gene expression by 2.20, 1.67, and 1.77-fold, respectively, in HCHF rats relative to the control rats. DATS-2 treatment significantly restored these downregulated expressions by 1.46 (p < 0.05), 1.41 (p < 0.001), and 1.31 (p < 0.01)-fold in PPAR-α, β, and γ, respectively, relative to HCHF rats. It is interesting to note that administration of DATS (DATS-1) at a low dose (1 mg/kg BW/rats/day) does not induce a marked change in PPAR-α, β, and γ expression levels (p > 0.05). Whereas SARO-treated rats exhibit a significant (p < 0.0001) upregulation of 2.32, 1.87, and 1.83-fold in PPAR-α, β, and γ expression levels, respectively, when compared to HCHF rats (Figures 9G–I).

### 3.12 DATS treatment reduces circulatory and hepatic lipid peroxidation products

**FIGURE 10:**
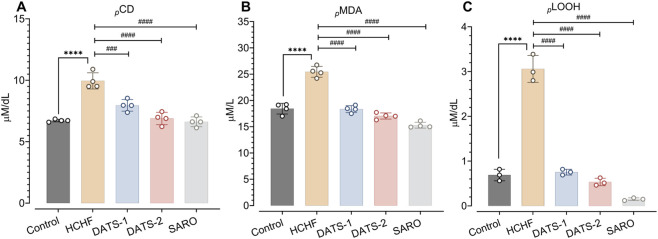
DATS and SARO protect plasma lipids against peroxidation. **(A)** Effect of DATS and SARO on the level of plasma conjugated diene (CD), **(B)** malondialdehyde (MDA), and **(C)** lipid hydroperoxide (LOOH) in HCHF-fed rats. Where *‘p’* stands for plasma lipid peroxidation products. Values are mean (μmol/dL) ± SEM from the plasma of four rats in each group. The statistical significance was evaluated by using one-way ANOVA followed by the Post Hoc Tukey-Kramer multiple comparisons test by using GraphPad Prism version 8.4.0. Significantly different from control at ****p < 0.0001. Significantly different from HCHF at ^####^p < 0.0001. Significantly different from HCHF at ^###^p < 0.001.

**FIGURE 11:**
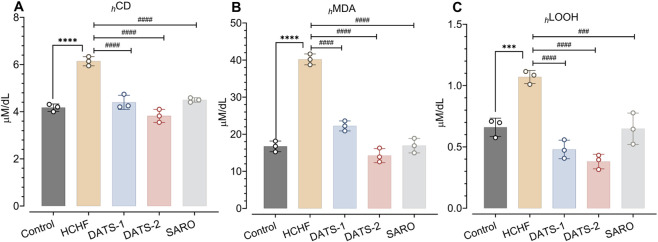
Effect of DATS and SARO treatment on hepatic lipid peroxidation products. **(A–C)** The level of hepatic CD, MDA, and LOOH in HCHF-fed rats after simultaneously treating with DATS and SARO for indicated time point. Where ‘*h’* stands for hepatic lipid peroxidation products. Values are mean (μmol/dL) ± SEM from the plasma of four rats in each group. The statistical significance was evaluated by using one-way ANOVA followed by the Post Hoc Tukey-Kramer multiple comparisons test by using GraphPad Prism version 8.4.0. Significantly different from control at ****p < 0.0001. Significantly different from Control at ***p < 0.001. Significantly different from HCHF at ^####^p < 0.0001. Significantly different from HCHF at ^###^p < 0.001.

Lipid oxidation by-products, *i.e.,* CD, LOOH, and MDA, are well-known markers identified as lipo-oxidative injury triggered by free radical action. Our results showed that plasma (_*p*_) _*p*_CD, _*p*_LOOH, and _*p*_MDA levels were markedly (p < 0.0001) elevated in HCHF group rats by 48.21%, 349.28%, and 38.02%, respectively, relative to the control group. Treatment with DATS significantly (p < 0.0001) deteriorated these elevated levels of _*p*_CD, _*p*_ *LOOH,* and _*p*_ *MDA,* with the maximum deterioration of 30.80%, 82.98%, and 33.08%, respectively, observed in the DATS-2-treated group relative to the HCHF group. Whereas treatment with the standard drug SARO also showed a remarkable protective property and diminished the oxidative stress significantly (p < 0.0001) by 33.53%, 95.16%, and 39.92% in _*p*_CD, _*p*_ *LOOH,* and _*p*_MDA, respectively, compared to HCHF rats (Figures 10A–C). Similarly, data from our *in vivo* experiments depicted that the consumption of HCHF diet resulted in an elevation of hepatic _*h*_CD, _*h*_ *LOOH,* and _*h*_MDA levels in HCHF rats by 46.89% (p < 0.0001), 59.70% (p < 0.0001), and 140.23% (p < 0.001), respectively, relative to the control rats. While 4 weeks treatment with DATS showed a marked reduction observed in hepatic _*h*_CD (p < 0.0001), _*h*_LOOH (p < 0.001), and _*h*_MDA (p < 0.0001) levels by 37.79%, 63.55%, and 64.64%, respectively, in DATS-2-treated rats when compared to HCHF rats, however, SARO-treated rats also revealed significant reduction of only 26.71% (p < 0.0001), 39.25% (p < 0.001), and 57.80% (p < 0.0001) in hepatic _*h*_CD, _*h*_ *LOOH,* and _*h*_MDA levels, respectively, relative to HCHF rats (Figures 11A–C).

### 3.13 Treatment with DATS regulates the enzymatic and non-enzymatic antioxidant status in liver tissue homogenate

**FIGURE 12:**
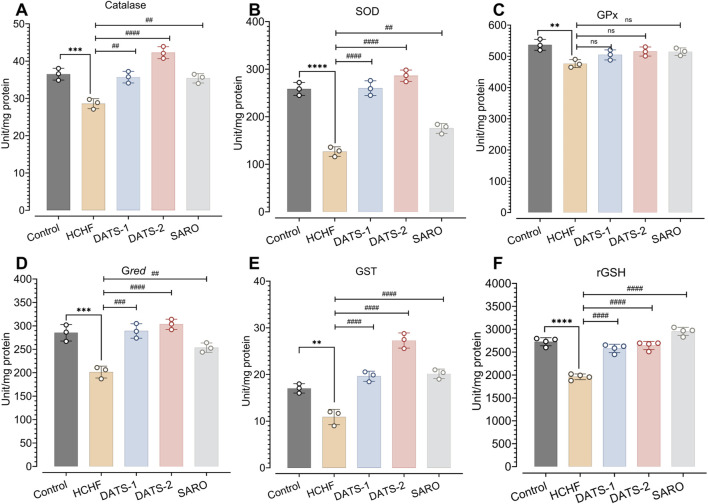
Effects of DATS and SARO treatment on hepatic enzymatic and non-enzymatic antioxidant systems in HCHF-induced cardiometabolic syndrome in rats. DATS and SARO improve hepatic enzymatic and non-enzymatic antioxidant system *i.e*., **(A)** Catalase, **(B)** SOD, **(C)** GPx, **(D)** G*red*, **(E)** GST, and **(F)** rGSH. The values are Mean ± SEM from homogenates/PMS fraction of liver of four rats in each group. One unit (U/mg protein) of catalase (CAT) activity is defined as μmoles of H_2_O_2_ decomposed/min/mg protein. One unit (U/mg protein) of superoxide dismutase (SOD) activity is defined as the amount of enzyme required to inhibit optical at 560 nm of chromogen produced by 50% in 1 min. One unit (U/mg protein) of glutathione peroxidase (Gpx) activity is defined as nmoles oxidized glutathione formed/min/mg homogenate protein. One unit (U/mg protein) of glutathione reductase (Gred) activity is defined as nmole NADPH oxidized/min/mg post mitochondrial supernatant (PMS) protein. One unit (U/mg protein) of GST activity is defined as the nmole of 1-chloro2,4-dinitrobenzene (CDNB) conjugate formed/min/mg PMS protein. The statistical significance was evaluated by using one-way ANOVA followed by the Post Hoc Tukey-Kramer multiple comparisons test by using GraphPad Prism version 8.4.0. Significantly different from control at ****p < 0.0001. Significantly different from control at ***p < 0.001. Significantly different from control at **p < 0.01. Significantly different from HCHF at ^####^p < 0.0001. Significantly different from HCHF at ^###^p < 0.001. Significantly different from HCHF at ^##^p < 0.01. Significantly different from HCHF at ^#^p < 0.05. Non-significant from HCHF at ^ns^p>0.05.

The antioxidant (enzymatic and non-enzymatic) status in MetS may play an important role in the progression and disease development that may result in an imbalance in the oxidant-antioxidant system. Therefore, it is important to assess the levels of GSH, a non-enzymatic antioxidant, as well as enzymatic antioxidants *i.e*., CAT, SOD, GPx, Gred, and GST in the liver. Our results depicted that the enzymatic activities of the liver CAT, SOD, GPx, Gred, and GST significantly declined by 21.75% (p < 0.001), 50.99% (p < 0.0001), 11.21% (p < 0.01), 29.51% (p < 0.001), and 36.05% (p < 0.01), respectively, relative to control rats. While DATS treatment exhibited a significant increase in CAT, SOD, GPx, Gred, and GST activities in a dose-dependent manner with the maximum restoration of 47.90% (p < 0.0001), 126.59% (p < 0.0001), 8.06% (p > 0.05), 50.53% (p < 0.0001), and 150.51% (p < 0.0001), respectively, in DATS-2 group, when compared to HCHF rats. However, rats treated with the standard drug SARO also ameliorated these enzymatic antioxidants up to 23.87% (p < 0.01), 38.67% (p < 0.01), 8.02% (p > 0.05), 25.87% (p < 0.01), and 84.94% (p < 0.0001), respectively, as compared to HCHF rats. On the other hand, the level of non-enzymatic antioxidant (GSH) was observed to be significantly reduced by 28.09% (p < 0.0001) in HCHF rats, relative to the Control rats. Whereas DATS administration exhibited marked enhancement in the GSH levels in a dose-dependent manner, with an enhancement of 35.08% (p < 0.0001) observed in DATS-2-treated rats, relative to HCHF rats. However, treatment with the standard drug SARO also improved the hepatic GSH content by 50.69% (p < 0.0001) compared to HCHF rats, which was even better than the effect observed in DATS-2 treated rats (Figures 12A–F).

### 3.14 Histo-architectural properties of the liver were restored after DATS treatment

**FIGURE 13:**
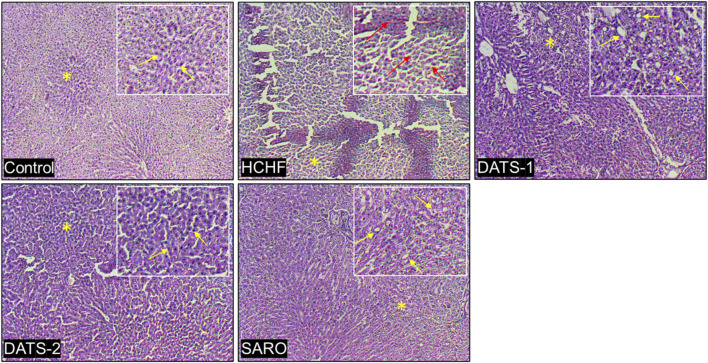
Hepatic histopathological investigation *via* hematoxylin and eosin staining. (Control): Control group showed uniform hepatocytes with vascular nuclei and abundant eosinophilic granule cytoplasm with distinct cell boundaries and well-maintained architecture with normal interstitial cell and vascularity; High-carbohydrate high-fat (HCHF): Histopathological investigation of HCHF administered rats showed degenerative changes in hepatocytes, their size, coarse granularity, decreased vascularity, and indistinct cell boundaries. The zoomed view presented in inset shows that hepatocytes were loosely packed with the existence of vacuolated spaces suggestive of dissolved cholesterol crystals (red arrows). Additionally, the accumulation of inflammatory cells was also reported; Diallyl trisulfide (DATS-1 and DATS-2): DATS administration showed improvement in histo-architecture in a dose-dependent manner when compared to HCHF with some smaller vacuolated spaces were reported in DATS-1 treated rats; Saroglitazar (SARO): Histological analysis of the liver from SARO-treated rats showed the restored morphology with some residual vacuolated spaces (yellow arrows), suggestive of presence of dissolved cholesterol crystals.

The hepatic histopathology of the control rats revealed uniform and normal hepatocytes with small vesicular nuclei and an abundance of eosinophilic granular cytoplasm with clear cell borders, as can be seen in Figure 13. The histoarchitecture is often well-maintained, with improved vascularity in regular interstitial cells. As expected, the HCHF group showed loosely packed smaller hepatocytes and smaller pyknotic or vesicular nuclei. The hepatic histoarchitecture was altered due to reduced vascularity, increased vacuolated spaces indicating cholesterol deposition, accumulation of inflammatory cells, cytoplasmic granularity loss, presence of needle like clefts suggestive of dissolved cholesterol crystals, pale or yellowish appearance, and cytoplasmic boundary diffusion. These histopathological features were markedly improved in DATS-1 treated rats however some smaller vacuolated spaces were still observed. Similarly, the histopathological features in DATS-2 treated rats were potentially improved and displayed marked hepatocyte proliferation, abundant eosinophilic cytoplasm, normally maintained vesicular nuclei, lack of vacuolated spaces left behind by dissolved cholesterol crystals, and distinct cell boundaries, regular interstitial cells, and vascularity with no signs of accumulation of inflammatory cells. Additionally, a micrograph of a liver portion from SARO-treated rats also revealed a significant increase in hepatic histoarchitecture with some smaller vacuolated spaces indicating residual cholesterol deposition.

## 4 Discussion

MetS corresponds to the collection of risk factors for CVD and T2DM, comprised of obesity, hypertension, glucose intolerance, dyslipidaemia, and IR, led by pathological conditions like oxidative stress and inflammation, which in turn lead to increased morbidity and mortality (Hirode and Wong, 2020; Silveira Rossi et al., 2022). Long-term MetS leads to multiorgan dysfunction and alters the structure of the heart, liver, kidney, and pancreas. An appropriate model organism that imitates all the above symptoms of MetS is required to evaluate possible pharmaceutical therapies for confronting MetS. Based on our previous *in silico* and *in-vitro* screening of naturally occurring OSCs for HMG-R (Ahmad et al., 2022), PCSK-9 (Ahmad et al., 2024), α-glucosidase, and α-amylase inhibitory potential (Ahmad et al., 2021), we selected DATS for the current *in-vivo* investigation. Hence, this article intends to evaluate the effectiveness of DATS against HCHF-diet-induced MetS in rats *via* targeting inflammatory markers, diabetic and lipid profile markers, and the expression of key genes that participate in the development and progression of MetS/ASVCD, i.e., HMG-R, PCSK-9, LDL-R, SREBP-2, HNF-1α, and PPAR (α, β, and γ).

It's well-renowned that feeding the HCHF diet induces dyslipidaemia in various animal models (Panchal et al., 2011; 2012; Ahmad et al., 2025). Our data also depicted that 4 weeks of administration of the HCHF diet exhibited similar characteristic symptoms of MetS as evidenced by a marked escalation in plasma TG, TGRLs, LDL-C, non-HDL-C, FBG, HBA1c, and insulin level. Increased levels of plasma TGRLs, LDL-C, and non-HDL-C were attributed to elevated activity of hepatic HMG-R in terms of mRNA transcription, translation, and functionality of the protein. This results in an increased level of cholesterol that is widely known to govern the processing of SREBP-2, resulting in excessive endogenous cholesterol and fatty acid production *via* HMG-R and fatty acid synthase (FAS), respectively (Ahmad et al., 2020; Waiz et al., 2022). It is well established that altered TG clearance and overproduction of TGRLs are responsible for carbohydrate-induced hypertriglyceridemia (Nabi et al., 2019). Our study also depicted similar results, as evidenced by a significant elevation in circulatory TG and LDL-C levels in the HCHF group. The subsequent treatment with DATS significantly decreased circulatory TC, TG, LDL-C, and VLDL-C levels and elevated HDL-C levels as compared to HCHF rats. These beneficial effects may be attributed to the effective inhibition of HMG-R activity and LDL-R-PCSK-9 interaction by DATS, which was recently established by our research group (Ahmad et al., 2022; 2024).

The hepatic HMG-R augments cholesterol synthesis restricts SERBP-2 processing, resulting in dysregulated cholesterol homeostasis (Goldstein and Brown, 2015; Ahmad et al., 2020). This SREBP, in turn, binds to the sterol regulatory element (SRE) present on multiple genes and activates a series of enzymes that participate in the cholesterol biosynthetic pathways, like HMG-R and FAS (Waiz et al., 2022; Ahmad P. et al., 2026). Our results also depicted that the hepatic HMG-R activity was increased by 1.64-fold in HCHF rats as compared to the control group. This elevated expression of HMG-R in HCHF rats was decreased by 1.36-fold after DATS treatment, as compared to HCHF rats, while treatment with SARO showed a 1.8-fold reduction in HMG-R activity which was better than the effect of DATS treatment. This is consistent with past studies that showed that the use of garlic extract supplements and OSCs significantly inhibits the escalation in serum lipid levels by limiting HMG-R activity (Sun et al., 2018; Ahmad et al., 2019; 2025).

On the other hand, the HCHF diet substantially elevated the FBG, HbA1c, and creatinine levels while decreasing the insulin level compared to the control rats. These data are strongly affirmed by earlier publications that revealed elevated FBG, HbA1c, and creatinine levels along with lower insulin levels in rats fed with HCHF diet (Panchal et al., 2011; Wong et al., 2018; Ahmad et al., 2025). Simultaneous DATS treatment markedly restored these parameters as compared to the HCHF group rats. Several reports confirm that feeding fructose leads to glucose intolerance and reduces insulin sensitivity in intact animals (D’Angelo et al., 2005). Furthermore, the improvement in these glycemic indices by DATS might be connected to the previous finding that DATS improves insulin levels by reducing reactive oxygen species (ROS)-induced loss of pancreatic β-cells through its potential antioxidant efficacy (Kuo et al., 2013; Ferguson et al., 2024).

Several reports have established the relationship between MetS and NAFLD’s presence, linked with cardiovascular remodeling and endothelial dysfunction. This liver disease includes simple steatosis, steatohepatitis, and cirrhosis. The initiation and progression of steatosis involves altered hepatic lipid metabolism, IR, hyperinsulinemia, and oxidative stress (Chen et al., 2022). The observed data showed an increased circulatory level of SGPT and SGOT in HCHF rats, which is in concordance with the prior reports (Poudyal et al., 2012; Ahmad et al., 2025). So, one of the best strategies to manage MetS-linked liver disease is to diminish overall oxidative stress and inflammation. We have observed a significant reduction in both elevated levels of SGOT and SGPT after DATS treatment owing to its potent antioxidant activity, along with the anti-inflammatory activity *via* averting the hepatic expression of pro-inflammatory cytokines.

Apart from cholesterol levels specifically LDL-C, PCSK-9 has now emerged as a ground-breaking therapeutic marker in the treatment of hypercholesterolemia (Goldstein and Brown, 2015; Waiz et al., 2022). The PCSK-9 alters LDL-C clearance by promoting LDL-R to lysosomal degradation *via* interacting with the EGF-A−domain of LDL-R, resulting in the unavailability of recycled LDL-Rs for further LDL-C clearance (Zhang et al., 2007; Alvi et al., 2017b). The expression of PCSK-9 is controlled by the action of SREBP-2 and HNF-1α (Xia et al., 2021; Waiz et al., 2022). As a result, establishing a pleiotropic pharmaceutical agent to reduce the levels of PCSK-9 and its transcriptional activator has become the desired signature in ASCVD care. Administration of the HCHF diet significantly increased the plasma PCSK-9 and HNF-1α levels in the current study, and this increase in PCSK-9 and HNF-1α levels could be directly linked to the elevated LDL-C level in the HCHF group. However, DATS treatment significantly decreased the escalated level of both PCSK-9 and HNF-1α. Current investigation suggests that the reduction in plasma PCSK-9 and HNF-1α protein levels may be linked to the restoration of LDL-C levels in rats treated with DATS.

Various reports suggested that HCHF consumption induces oxidative stress-mediated metabolic disturbances that causes cellular insult and formation of oxidized-LDL-C, resulting in initiating cellular inflammatory response in the etiopathology of MetS (Alvi et al., 2017b; Zhang et al., 2017). The accumulation of proinflammatory cytokines (e.g., IL-1α, IL-1β, IL-6, and TNF-α) and chemokines like MCP-1 collectively initiates an inflammatory cascade (Ahmad et al., 2020). The increase in these pro-inflammatory cytokines in the current study is also well supported by previous studies conducted in high carbohydrate or fructose-rich diet animals (de Almeida-Souza et al., 2021). It is well established that TNF-α was strengthened further by the amplified cytokines and CRP concentrations in plasma. The CRP, a circulatory hepatic protein, and MCP-1 are well-known biomarkers for systemic inflammatory response and tissue injury. Elevated levels of CRP are also a risk factor for IR and MetS as well as an independent determinant for future cardiovascular events (Koziarska-Rościszewska et al., 2021). The CRP has been documented to stimulate MCP-1 levels in endothelial cells as well as its receptor, chemokine receptor type 2, in monocytes (Sproston and Ashworth, 2018). Recently, Wang et al. reported an enhanced MCP-1 level in high fructose diet-administered rats (Wang X. et al., 2023). Similarly, our results also depicted that circulatory inflammatory biomarkers like IL-1α, IL-1β, TNF-α, CRP, and MCP-1 levels were markedly elevated in HCHF-diet-induced rats, which was in agreement with the formerly available reports (Antunes et al., 2020; de Almeida-Souza et al., 2021).

Treatment with DATS significantly lowered the elevated inflammatory markers, with the maximum reduction observed in DATS-2 and standard drug SARO-treated rats, when compared to the HCHF group. The reversal of inflammatory cascades following DATS intervention may be attributed to the reduction of ROS-induced expression of the transcription factor, nuclear factor-kappa B, and concurrent transcription of its downstream inflammatory genes (Akhter et al., 2021). These results were well corroborated by a previously published report showing that DATS and SAC decrease the gene expression of cytokines related to the proinflammatory responses, *e.g.,* TNF-α, IL-1α, IL-β, and MCP-1 (Rodrigues and Percival, 2019). The declining level of pro-inflammatory mediators by DATS might have enabled insulin receptor substrate phosphorylation in MetS. These beneficial events can restore the inhibitory effect on insulin signaling pathways, primarily fatty acid enzymes, glucose uptake, and diminish the synthesis of FFA. However, in our investigation, the reduction of such inflammatory mediators can also be linked to a decline in vascular PCSK-9 levels, since inflammation itself promotes the stimulation of PCSK-9 expression (Feingold et al., 2008; Alvi et al., 2017a). A recently published report has also established the positive correlation of PCSK-9 with ApoCIII, CXCL2, and MCP-1 in T2DM and T2DM with dyslipidemia subjects suggesting a possible interplay between lipid metabolic, diabetic, and inflammatory pathways in MetS (Waiz et al., 2026). On the other hand, gut microbiota has been extensively associated with the pathogenesis of MetS, NAFLD, and associated hepatic inflammation (Sanz et al., 2015; Gao, 2024). Any imbalance in gut microbial load, also termed as dysbiosis, leads to the weakening of the gut barrier, increasing gut permeability and releasing bacterial products like lipopolysaccharides into circulation. This leaky gut triggers chronic inflammation, insulin resistance, and hepatic fat accumulation, linking gut dysfunction directly to liver diseases (MAFLD/NAFLD) and metabolic dysfunction (Divya et al., 2023; Gao, 2024). Given that DATS is an orally administered OSC, its profound anti-inflammatory and hepatoprotective effects observed in our study may, in part, be mediated through modulation of gut microbiota composition and function. However, future studies should investigate the impact of DATS on the gut-liver axis, as alterations in microbial metabolites (e.g., short-chain fatty acids) could contribute to the suppression of hepatic inflammatory cytokines and the regulation of cholesterol metabolism observed here.

The development of MetS is known to be significantly influenced by oxidative damage, which arises from an imbalance between ROS/reactive nitrogen species (RNS) and the antioxidant system (Dash et al., 2025). The persistent intake of the HCHF diet has been proven to trigger oxidative stress, which can be characterized by reduced FRAP and HDL-linked PON-1 activity (Alvi et al., 2017a). Our data showed a similar pattern of FRAP and PON-1 activity in the HCHF group rats. Treatment with DATS significantly enhanced the circulatory FRAP and PON-1 functionality in a dose-dependent manner, which can be further linked with improved plasma HDL-C levels. The restoration of plasma levels of FRAP and PON-1 activity may be attributed to the robust antioxidant properties of DATS (Kuo et al., 2013). The above data are well justified by a few reports that displayed garlic extract or garlic-derived OSCs; SAC, NAC, and DATS decreased the lipid peroxidation in chemical or diet-induced oxidatively stressed rats (Uddandrao et al., 2016; Ahmad et al., 2025).

To explain the molecular mechanism responsible for the pronounced hypolipidemic impact, we also evaluated the influence of DATS on the gene expression levels related to cholesterol homeostasis, *viz.*, PCSK-9, LDL-R, HMG-R, SREBP-2, and HNF-1α. The results showed that the expression of HMG-R, PCSK-9, and its transcriptional activators, SREBP-2 and HNF-1α, was significantly upregulated, while the LDL-R expression was comparatively downregulated in HCHF rats relative to the control rats. Similar outcomes were also noted in earlier research (Alvi et al., 2017a; 2017b; Ahmad et al., 2025). The increased plasma PCSK-9 level in the present investigation can also be linked to this elevation in PCSK-9 mRNA expression in HCHF group. Additionally, a shortage of functional hepatic LDL-R due to PCSK-9-directed LDL-R destruction limits receptor-mediated cholesterol intake into the cells, which in turn increases HMG-R expression to compensate the limited cholesterol reserves inside the cells. This could account for an upregulation in hepatic HMG-R transcription and enzymatic activity. It has been demonstrated that statins, the most popular HMG-R inhibitors and effective lipid-lowering drugs over the past 7 decades, increase PCSK-9 expression by activating SREBP-2. This limits their therapeutic efficacy when it comes to total LDL-C clearance and raises concerns about the development of alternative PCSK-9 inhibitors (Ahmad et al., 2020; Waiz et al., 2022). Unlike statins, DATS treatment substantially lowered the HMG-R and PCSK-9 expression by downregulating HNF-1α, an obligatory trans-activator for PCSK-9 gene expression, which interacts with the PCSK-9 promoter and supports its overexpression (Waiz et al., 2022). In contrast, as compared to the HCHF group, an upregulation of LDL-R mRNA expression was observed in DATS-treated group. This reduction in hepatic PCSK-9 expression by DATS led to an escalation in the number of surface LDL-R that was available to remove atherogenic LDL-C particles, which was well justified by our data showing significant upregulation in LDL-R gene expression and limiting the hepatic HMG-R expression. There are currently a handful of natural substances that have been shown to have dual inhibitory action against the expression of PCSK-9 and HMG-R (Alvi et al., 2017a; 2017b; Ahmad et al., 2020; 2024; 2025).

High fructose diet-induced inflammation ultimately induces marked changes in lipid metabolism, and similar studies have shown a straight linkage between inflammation and PCSK-9 expression (Dong et al., 2015). Taking into consideration the destructive role of inflammatory cascades in ASCVD and MetS, we also investigated how HCHF-diet consumption affected the inflammatory cytokines/chemokines expression. Here, we found that HCHF rats exhibited significantly higher hepatic inflammatory cytokines/chemokines mRNA expression i.e., IL-1β, IL-6, TNF-α, CXCL-1, CXCL-2, and MCP-1. These results are corroborated by increased levels of circulatory cytokines in rats with MetS/hyperlipidemia brought on by a high fructose diet and in clinical contexts (Fahmy et al., 2022; Waiz et al., 2023). Treatment with DATS and SARO significantly reduced the altered expression of inflammatory cytokines/chemokines mRNA. Based on our data, the cytokine and chemokine’s respective plasma levels were in good agreement with the downregulation in their mRNA expression. DATS may have achieved this inflammatory cascade recovery by hindering ROS-induced NF-kB activation (Lingappan, 2018). According to the aforesaid results, it can be summarized that expression of hepatic PCSK-9 was downregulated in the HCHF rats after DATS treatment downregulated the inflammatory cascades.

According to several reports, PPARs influence gene expression along with important metabolic pathways involved in energy and lipid metabolism. Therefore, they are considered crucial therapeutic targets for the treatment of MetS (Bougarne et al., 2018). In accordance with our gene expression study, PPAR-α, -β, and -γ mRNA expression was significantly downregulated in HCHF-diet-consuming rats relative to the control group. Our findings are in agreement with those of other previously published reports (Farag et al., 2020; Kumari et al., 2023). However, the PPAR-α, −β, and −γ expression was improved after subsequent DATS and standard drug SARO treatment, with maximum restoration observed in SARO-treated rats. Restoration in the expression of PPAR-α, −β, and −γ by DATS was observed to be less than standard drug. This DATS-induced overexpression of PPARs can be directly linked to decreased PCSK-9 activity, lipid levels, and insulin sensitivity, all of which contribute to improving overall blood cholesterol, diabetic profile, and Mets.

On the other hand, oxidative stress, which impacts several macromolecules, such as proteins, nucleic acids, and lipids, can be triggered by a number of reasons, including lifestyle choices, exercise, stress, and environmental stimulants (Nabi et al., 2018; 2020). Among these, lipids are easily encountered by circulatory ROS to produce various modified adducts, *i.e*., CD, LOOH, and MDA (Ayala et al., 2014). Therefore, the enhanced circulatory lipid peroxidation level indicates cell membrane damage in some organs or tissues (Ayala et al., 2014). Consistent with these reports, our results revealed that HCHF-induced diabetic rats had higher levels of hepatic and circulatory lipid peroxidation products than the control group. The elevation in lipid peroxidation products in Mets is strongly linked with the substantial reduction in circulatory total antioxidant as well as HDL-linked PON-1 functionality (Dornas and Silva, 2024), which was again well corroborated with our findings that depicted a marked reduction in FRAP and plasma PON-1 activity in HCHF-diet stimulated MetS in rats. The amount of these lipid peroxidation products was considerably decreased by concurrent administration of DATS and the standard drug SARO. Our results are consistent with earlier studies showing how natural ingredients can prevent lipid peroxidation events (Jahan et al., 2022).

However, increased ROS production in HCHF-diet-induced MetS results in enzymatic and non-enzymatic antioxidant imbalance (Ibitoye and Ajiboye, 2018). Enzymatic antioxidants are important in scavenging free radicals. A diet-induced MetS in rat model showed a drop in major SODs and an increase in oxidative stress and endothelial dysfunction, suggesting that the antioxidant defense mechanism is disrupted in MetS (Roberts et al., 2006). Superoxide radicals are scavenged by SOD to create hydrogen peroxide (H_2_O_2_). This H_2_O_2_ is neutralized by CAT/GSH, or it promotes the production of high ROS (Ighodaro and Akinloye, 2018). In the presence of free radicals, the enzymatic activity of CAT declines, resulting in the buildup of lipid hydroperoxides and H_2_O_2_, hence exacerbating tissue damage (Ighodaro and Akinloye, 2018; Hashim et al., 2019). Additionally, we noticed a marked decrease in the liver’s CAT, SOD, Gpx, G*red*, GST, and GSH levels, which ultimately concluded a lower *in-vivo* antioxidant level that is insufficient for defense against the oxidative stress caused by the HCHF diet. However, the hepatic antioxidant activity was significantly increased by the concurrent administration of DATS and SARO. While the Gpx activity remains non-significant after the treatment with both the drugs, DATS and SARO. However, the GSH level was also restored in the DATS and SARO-administered groups, which can be due to reduced G*red* activity and minimal supply of NADPH. Which additionally supports the potent free radical scavenging property of the DATS (Kuo et al., 2013). On the other hand, the data from recent reports has established that hydrogen sulfide (H_2_S) is a vital gaseous signaling molecule and its deficiency is closely linked to the pathogenesis of MetS and its associated vascular complications, including endothelial dysfunction, insulin resistance, and atherosclerosis (Wang M. et al., 2023; Smimmo et al., 2024; Zampas et al., 2025). The deficiency of H_2_S under the influence of HCHF-induced MetS is attributed to the downregulated expression and enzymatic activity of cystathionine-γ lyase (CSE), an enzyme responsible for H_2_S production in endothelial cells (Smimmo et al., 2024). Moreover, H_2_S deficiency has also been linked to the suppression of 3-mercaptopyruvate sulfur transferase (MPST), a cysteine-catabolizing enzyme that generates pyruvate and H_2_S (Zampas et al., 2025). Altered CSE/MPST/H_2_S pathways leads to compromised endothelium-dependent relaxation and increased vascular stiffness, which are early steps in atherosclerosis (Smimmo et al., 2024). Therefore, restoration of H_2_S bioavailability using donors or precursor supplementation shows therapeutic potential to reverse these cardiometabolic abnormalities. H_2_S donors, particularly Erucin, sodium hydrosulphide, and GYY4137, have shown profound beneficial impact against vascular dysfunction *via* amelioration of CSE/MPST, Ca^2+^/CaMKK2/AMPK and PI3K/AKT pathways, ATP/cAMP, and cGMP system (Chen et al., 2017; Smimmo et al., 2024; Zampas et al., 2025). In this context, DATS, a promising garlic-derived OSC, may act, at least in part, *via* amending the compromised H_2_S levels through endogenous CSE/MPST pathways.

Furthermore, according to our histopathological examination, the rats fed with the HCHF diet showed marked inflammation and degenerative changes in liver, including changes in size, shape, granularity, increased vacuoles indicative of dissolved cholesterol crystals, vascularity, striation, hyalinization, cell borders, and areas of infarction as compared to the control rats. The aberrant histoarchitecture was substantially returned to nearly normal texture after treatment with DATS and SARO. Unlike SAC and SEC, which are water-soluble, DATS possesses a unique trisulfide backbone and higher lipophilicity, which might facilitate deeper membrane penetration and broader cellular interactions. This structural advantage might translate into enhanced modulation of key cholesterol, redox homeostasis, and inflammatory pathways. These pleiotropic mechanistic effects of DATS emphasize its potential as comprehensive and potent therapeutic candidate for managing HCHF-induced MetS. Though this study provides preliminary therapeutic potential of DATS against HCHF diet-induced MetS *via* targeting various biochemical and molecular markers, further studies are required with larger numbers of animals, long duration to mimic chronic disease conditions, and different models of MetS to make more generalized conclusions.

## 5 Conclusion

The above-discussed collective findings indisputably demonstrate that DATS effectively mitigates all major pathological features of MetS, including redox imbalance, dyslipidemia, hyperglycemia, inflammation, and hepatic dysfunction. This is the first comprehensive *in-vivo* study on HCHF-diet-induced MetS to show that DATS regulates key cardiometabolic risk factors by targeting oxidative stress, inflammation, diabetic biomarkers (FBG, insulin, HbA1c), PCSK-9 *via* modulation of HNF-1α, and gene expression pathways related to lipid and glucose metabolism (HMG-R, LDL-R, SREBP-2, and PPARs). Conclusively, DATS, a dietary garlic-derived OSC, exerts profound therapeutic efficacy against MetS, hypercholesterolemia, and diabetes. Since we used a single rat model to test our hypothesis, further mechanistic, long-term, and comparative studies with different OSCs in various murine models of MetS are warranted to make a generalization about such pharmacological impact of DATS.

## Data Availability

The original contributions presented in the study are included in the article/Supplementary Material, further inquiries can be directed to the corresponding authors.
