# Supplementary material for: Diallyl trisulfide attenuates metabolic syndrome via integrated modulation of PCSK-9/LDL-R axis, redox homeostasis, and inflammatory cytokine networks in high-carbohydrate high-fat (HCHF) diet-fed rats
Source: Front Pharmacol. 2026 May 29;17:1781950. doi: 10.3389/fphar.2026.1781950 (PMC13259818; doi:10.3389/fphar.2026.1781950)
Supplement: Supplementary file 1 [file Table1.docx]

Supplementary Material

# Supplementary Data

**Supplementary Table 1.** Oligonucleotide primers used for gene expression study.

| **S. No.** | **Gene Name** | **Forward Sequences (From 5’→3’)** | **Reverse Sequences (From 5’→3’)** |
| --- | --- | --- | --- |
|  | SREBP-2 | GGGGCCATTCTGACCACAA | ACCCCCAGGCACTTGCTTA |
|  | PSCK-9 | TGATCGAATTGAGGCCATAGG | CCCTCACCCCCAAATGC |
|  | LDLr | ACGGGCTGGCGGTAGACT | GGAAACAGTGCCCGGAACT |
|  | HNF-1α | TTCACTAACACGGGTGCCTCTA | TGATGACTGGCACGCTCTGT |
|  | HMG-R | CGTGCTGAGCAGCGACAT | TGTACAGGATGGCGATGCA |
|  | IL-1β | TGACAGACCCCAAAAGATTAAGG | CTCATCTGGACAGCCCAAGTC |
|  | IL-6 | ACTTCTCCCCCACCGTATCC | GAGGAGAGGAACCAGGAGGAA |
|  | MCP-1 | CTGTCTCAGCCAGATGCAGTTAAT | AGCCGACTCATTGGGATCAT |
|  | CXCL-1 | CAGACAGTGGCAGGGATTCA | CCTGGCGGCATCACCTT |
|  | CXCL-2 | CAGGGGTTGTTGTGGCCA | AGCTCTGGATGTTCTTGAAGTCAA |
|  | TNF-α | GGTCCCAACAAGGAGGAGAAGT | CTGGGCCATGGAACTGATG |
|  | PPAR-α | ACGATGCTGTCCTCCTTGATG | GCGTCTGACTCGGTCTTCTTG |
|  | PPAR-β | GCCGCCCTACAACGAGATCA | CCACCAGCAGTCCGTCTTTGT |
|  | PPAR-γ | CCCTTTACCACGGTTGATTTCTC | GCAGGCTCTACTTTGATCGCACT |
|  | β-actin | GCGCGGCTACAGCTTCA | TCTCCTTAATGTCACGCACGATT |

**Abbreviations:** CXCL, chemokine ligand; HMG-R, 3-hydroxy-3-methylglutaryl coenzyme-A; HNF-1α, hepatic nuclear factor-1 Alpha; IL-1β, interleukin-1β; IL-6, interleukin-6; LDL-R, low-density lipoprotein receptor; MCP-1, monocyte chemoattractant protein-1; PPAR, peroxisome proliferator-activated receptors; PSCK-9, proprotein convertase subtilisin/kexin type 9; SREBP-2, sterol-regulatory element-binding protein-2; TNF-α, tumor necrosis factor-α.
